# Understanding the role of cybersecurity in the internet of things within the health sector: an empirical study

**DOI:** 10.3389/fpubh.2026.1851079

**Published:** 2026-07-17

**Authors:** Ruba A. Alnajim, Ali A. Alkhalifah

**Affiliations:** Department of Information Technology, College of Computer, Qassim University, Buraydah, Saudi Arabia

**Keywords:** awareness, chronic diseases, cybersecurity, healthcare, internet of things, privacy, trust

## Abstract

**Introduction:**

The digital healthcare environment is rapidly evolving, driven by the Internet of Things (IoT), which offers benefits like early diagnosis, monitoring, and cost reductions. However, this growth heightens the risk of cyberattacks, raising crucial concerns about patient safety, data breaches, and the necessity of trust for technology adoption. This study explores patient acceptance of digital health technologies for chronic disease management.

**Methods:**

A research framework was developed combining Protection Motivation Theory (PMT) and the Task–Technology Fit (TTF) model. This framework was augmented by incorporating comprehensive user-centric, behavioral, and technological dimensions. Data from 603 participants were analyzed using a hybrid methodology of Structural Equation Modeling (SEM) and Artificial Neural Networks (ANNs). The ANN was deployed to capture complex, non-linear relationships among variables, overcoming the linear limitations of traditional SEM to rank predictor importance with higher accuracy.

**Results:**

Findings reveal that while cybersecurity concerns exist, they do not actively deter technology adoption; rather, they are statistically secondary to immediate health benefits and system usability, especially for patients with chronic diseases. Factors including functionality, information accuracy, trust, privacy, and training did not significantly influence IoT adoption, while awareness, perceived vulnerability, perceived severity, innovation, risk, and compliance exerted minor effects.

**Conclusion:**

This study advances digital health literature by providing a novel, dual-theoretic framework (PMT-TTF) validated through machine learning, demonstrating that utilitarian health value overrides security anxieties in chronic care contexts. The findings offer practical insights for healthcare providers and developers to prioritize user-centric design alongside robust security protocols.

## 1 Introduction

The Internet of Things (IoT) has transformed healthcare by enabling continuous home monitoring for chronic conditions like diabetes and heart disease (1). These smart devices track biomarkers in real time, provide early warning alerts, and improve medication adherence (2). By reducing hospital readmissions and institutional costs, the global healthcare IoT market is projected to reach $342.57 billion (3). However, this rapid expansion vastly increases the healthcare cyber-attack surface. Moving medical devices onto consumer-managed home networks creates severe vulnerabilities that bypass traditional hospital firewalls (4). Healthcare has consequently become a primary target for cybercriminals, as seen in the U. S. with 3,800 major breaches exposing 283 million records over 10 years (5). Unlike standard corporate data breaches, compromising an IoT medical device such as a connected pacemaker or insulin pump can cause immediate, life-threatening physical injury (6).

This tension is highly critical in Saudi Arabia, where digital health transformation is a core pillar of Saudi Vision 2030. Under the Health Sector Transformation Program, the Ministry of Health has deployed large-scale platforms like the SEHA Virtual Hospital and the Sehhaty app to transition patient care to digital ecosystems (7). In addition, hospitals are increasingly implementing AI-based diagnostics and IoT medical devices to monitor and facilitate early detection of chronic diseases like cancer and diabetes. However, these rapid rollouts often outpace public digital literacy. In developing digital economies like Saudi Arabia, up to 75% of surveyed e-health users have reported unauthorized data compromises (8), exposing vulnerable chronic patients to unique security risks.

Despite these dangers, empirical literature remains fragmented regarding how cybersecurity anxieties shape patient behavior. Existing studies often treat “security” as a monolithic deterrent (7). This overlooks the unique trade-off behavior of chronic patients, who must constantly weigh the immediate, life-saving utility of an IoT device against the invisible threat of a data breach. Furthermore, standard utilitarian models like Task-Technology Fit (TTF) ignore security fears, while threat-centric frameworks like Protection Motivation Theory (PMT) overlook whether the technology satisfies the user’s practical daily needs (9).

This study introduces an integrated framework synthesizing TTF and PMT. Survey data from 603 chronic disease patients who are aware of IoT health monitoring systems. Then follows a two-stage methodological process designed to address the identified research gap on patients’ cybersecurity experiences in the context of IoT. First, the research instrument is developed through a structured online survey methodology. Specifically, the survey items are generated based on relevant literature and refined through instrument validation, ensuring content clarity and measurement suitability for the constructs under investigation. Second, the study proceeds with sampling and data collection, where participants meeting the eligibility criteria complete the questionnaire through an online platform. Finally, the analytical workflow applies a hybrid approach combining PLS-SEM and Artificial Neural Networks (ANN). While PLS-SEM is used to test the hypothesized relationships between key variables and evaluate measurement and structural validity, ANN is further employed to capture potential nonlinear patterns and enhance predictive insights regarding how cybersecurity-related factors relate to anxiety-driven patient behavior.

The empirical findings reveal that while cybersecurity concerns exist, they do not actively deter technology adoption; instead, they are statistically secondary to immediate health benefits and system usability. While functionality, information accuracy, trust, privacy, and training operated merely as basic hygiene factors, variables such as awareness, perceived vulnerability, perceived severity, personal innovativeness, risk, and regulatory compliance exerted significant direct effects. Ultimately, this study demonstrates that utilitarian health value overrides security anxieties in chronic care, providing a blueprint for balancing user-centric medical design with robust compliance protocols.

This article is structured into eight sections: Section 2 reviews the existing literature and theoretical background, Section 3 describes the underlying model, which leads to the hypotheses, Section 4 describes the research methodology, Sections 5 and 6 discuss and analyze the results, and Sections 7 and 8 summarize the contributions and conclusions.

## 2 Literature review and theoretical background

### 2.1 The internet of things (IOT) in healthcare

The Internet of Things (IoT) has emerged as a disruptive technological paradigm capable of fundamentally altering how human agents interact with physical and clinical environments (1). Within contemporary healthcare, the IoT architecture primarily facilitates continuous remote patient monitoring (2). Optimizes institutional workflows (10) and dynamically captures longitudinal data for chronic disease management (11). This ecosystem spans a diverse continuum, ranging from consumer-grade wearables to highly sophisticated, implantable medical devices.

In mobile health contexts, non-invasive edge devices—including smartwatches, smart patches, and textile biometric sensors—allow for the continuous telemetry of vital signs such as electrocardiograms (ECG), blood pressure, and respiration metrics (12). Concurrently, high-risk invasive devices—such as network-enabled pacemakers, automated insulin pumps, and subcutaneous glucose monitors—provide critical, localized therapeutic interventions (13).

### 2.2 Key challenges: security and privacy

Despite all the previously mentioned benefits, prior research dug deep into challenges and issues facing the IoT adoption, specifically in a sensitive environment such as the healthcare industry, where data involves personal information, and Cyberattacks may result in fatal consequences. A recent research done by Al Khatib et al. (14) identified security and privacy problems, as highly sensitive health data is susceptible to breaches, unauthorized access, and device compromise. Moreover, scholars of Naresh et al. (15) point out that device makers or vendors often overlook interoperability and standards, which limit seamless data interchange and large-scale implementation. Additionally, authors of Al Khatib et al. (14), see Technical barriers, particularly power and scalability, remain as a core issue, especially for continuous monitoring. Others Almotairi (16) emphasized the significance of human and organizational aspects, as user acceptance, governance, and legal frameworks profoundly influence adoption. Overall, this determines the necessity for more research to identify barriers and facilitators for IoT adoption among patients to maximize benefits.

### 2.3 Factors influencing IoT adoption in the healthcare sector

Extant literature confirms that the adoption trajectory of healthcare IoT is multi-dimensional, shaped by a confluence of technical, human, regulatory, and security determinants. A recent systematic literature review (SLR) synthesizing the historical landscape of this domain revealed prominent empirical gaps (17). Adoption models have been dominated by baseline cognitive constructs such as perceived usefulness, user attitudes, and interpersonal trust (18). For instance, clinical trust in automated systems (e.g., smart infusion pumps) directly accelerates adoption, thereby eliminating manual dosing anomalies. Similarly, systemic characteristics like network connectedness and device reliability serve as foundational prerequisites for embedding these solutions into wider Electronic Health Record (EHR) frameworks (19). However, the transition of these devices into the patient’s domestic space has heightened the importance of robust security assurances and strict regulatory compliance (such as HIPAA mandates), which dictate both organizational and individual trust (20). Despite these insights, the systematic review highlighted a persistent blind spot: empirical studies explicitly isolating *cybersecurity behavior* and *threat-coping mechanisms* among patients remain scarce. Most models treat security as a passive environmental variable rather than an active, cognitive decision-making process. This theoretical and empirical gap directly motivates the current investigation.

### 2.4 Theoretical foundation

#### 2.4.1 Protection motivation theory (PMT)

Protection Motivation Theory (PMT) provides a robust psychological framework for evaluating how individuals alter their behavioral intentions when exposed to environmental risks or information security threats (21). Originally developed to explain health-related fear appeals, PMT has become a cornerstone framework within Information Systems (IS) security literature to predict compliance, risk-mitigation strategies, and technology avoidance behaviors (22, 23). The architecture of PMT posits that individuals undergo two distinct cognitive processes when confronted with a threat: Threat Appraisal and Coping Appraisal (24). Threat appraisal evaluates the cognitive weight of an impending danger through two distinct variables: Perceived Severity (PS), the perceived seriousness of the threat’s consequences, and Perceived Vulnerability (PV), the perceived likelihood that the threat will directly impact the individual. Within technology acceptance literature, PMT has consistently demonstrated superior predictive power over traditional, purely utilitarian frameworks. For example, Park et al. (25) integrated PMT with the Technology Acceptance Model (TAM) to study AI service adoption, concluding that threat-defense variables were more powerful predictors of user intent than baseline ease-of-use metrics. Similarly, hybrid implementations combining PMT with other behavioral frameworks have successfully explained adoption vectors in electronic authentication services (26), contactless financial transactions during health crises (27), data-sharing applications (28), and mHealth platforms for vulnerable aging demographics (29). Given that the healthcare IoT exposes chronic patients to parallel, dual-threat landscapes (simultaneous health vulnerabilities and digital privacy threats), this study extracts the threat appraisal sub-constructs (PS and PV) to explicitly capture how risk perceptions alter adoption trajectories.

#### 2.4.2 Task–technology fit (TTF) model

The Task–Technology Fit (TTF) model operates on the foundational premise that information systems positively impact performance and utilization only when their structural features closely align with the practical tasks the user must execute (30). The model is explicitly anchored by two baseline dimensions: Task Characteristics (the operational actions required to convert inputs to outputs) and Technology Characteristics (the specific tools or functional attributes applied to fulfill those requirements). In contemporary information security contexts, TTF is widely utilized to assess whether security architectures act as functional enablers or operational bottlenecks that slow user workflows (31). Outside of security compliance, integrated TTF frameworks have explained user behaviors across wearable devices (32) and complex care delivery platforms for multi-morbid populations (33). While some studies note that applying TTF in isolated clinical settings can yield inconsistent results (34) it remains an essential predictor of utilization when explicitly married to domain-specific tasks. In chronic care contexts, the essential task is continuous, real-time health management, while the “technology” is the IoT framework capturing vital biometric metrics.

If an IoT device yields inaccurate data, the task-technology alignment collapses, triggering severe consequences: incorrect pharmaceutical adjustments, false alarms that induce acute anxiety, or catastrophic failures to signal critical clinical events. Because this task-technology alignment directly dictates patient safety and quality of care, evaluating user trust in the device’s functional capacity is essential.

#### 2.4.3 Why integrate PMT and the TTF model?

While both PMT and TTF offer powerful independent explanations for user behavior, relying on either model in isolation creates an epistemological blind spot when evaluating healthcare IoT adoption. TTF is an inherently rational, utilitarian model. It assumes that if a technology fits a task, adoption will seamlessly follow. It entirely fails to account for emotional friction, privacy anxiety, or the psychological paralysis caused by perceived cybersecurity vulnerabilities (35). Conversely, PMT excels at capturing fear appeals and defensive motivations, but it completely ignores the functional utility of the tool. A patient may feel highly threatened (high PMT), but if a device does not technically fit their daily clinical routine (low TTF), PMT alone cannot accurately map their adoption behavior (36). Integrating PMT and TTF establishes a balanced cognitive framework that harmonizes psychological risk mitigation with practical functional utility (35). Research confirms that both frameworks exhibit reduced variance and heightened statistical reliability when combined with a complementary model (36). By unifying these two domains, our integrated framework captures the realistic, complex trade-offs chronic patients make when balancing the clinical necessity of health tracking against the background anxiety of cybersecurity threats.

While models such as the Health Belief Model (HBM), Technology Threat Avoidance Theory (TTAT), Theory of Reasoned Action (TRA), and the Expectation-Confirmation Model (ECM) provide isolated insights into behavioral intent, they possess systemic limitations for this research question. Frameworks like HBM and TTAT focus heavily on preventive behavioral avoidances and fear appeals, yet they lack structural indicators to evaluate operational alignment with day-to-day functional clinical requirements (the “fit” dimension). Conversely, purely transactional or post-adoptive models like TRA and ECM assume baseline consumer utility or cognitive intention while completely omitting high-stakes physical safety risks and security threat appraisals. The deliberate, synergistic integration of PMT and TTF overcomes these limitations, establishing a balanced framework that evaluates data accuracy and clinical task performance alongside acute psychological anxieties over unauthorized medical edge data manipulation.

#### 2.4.4 Critical synthesis of prior literature

To map the evolutionary landscape of healthcare IoT adoption and contextualize the current study’s theoretical integration, Table 1 provides an analytical, critical evaluation of prior foundational studies.

**Table 1:** Critical synthesis of prior literature on healthcare IoT adoption.

Study	Theoretical core	Primary focus/Context	Methodological approach	Limitations
Park et al. (25)	PMT + TAM	AI Avatar Services & Consumer Trust	Structural Equation Modeling (SEM)	Overemphasized abstract cognitive intent; failed to address high-stakes clinical tasks or cyber-physical safety risks.
Kim and Kyung (26)	VAM + PMT + TPB	Next-Gen Electronic Authentication	Hybrid Regression	Evaluated transactional security behaviors but isolated them from long-term, life-sustaining medical dependencies.
Chiu et al. (29)	PMT + TR	Older Adults’ mHealth App Adoption	Traditional SEM	Ignored the dynamic fit between evolving patient clinical conditions and complex hardware characteristics.
Chang et al. (32)	TAM + TTF	Consumer Wearable Health Devices	PLS-SEM	Assumed adoption was purely a function of utility and fit; omitted privacy concerns and threat anxiety entirely.
Hwang et al. (31)	TTF + POF	Information Security Policy Compliance	Covariance-based SEM	Focused exclusively on organizational compliance; failed to capture individual patient-side psychological trade-offs.
Current Study	Integrated PMT + TTF	Iot Chronic Patient Cybersecurity Behavior (Saudi Arabia)	Hybrid SEM-Artificial Neural Networks (ANN)	*Addresses prior gaps by cross-examining task-technology alignment alongside threat appraisals using advanced non-linear machine learning.*

The structural breakdown provided in Table 1 is vital for understanding the critical empirical gaps this study targets. As synthesized above, prior academic inquiries have consistently faced a conceptual dichotomy: researchers either focused exclusively on abstract cognitive intent (e.g., PMT + TAM paradigms) while ignoring hands-on, high-stakes medical monitoring tasks, or focused entirely on technical, organizational policy compliance (e.g., TTF implementations) while ignoring individual patient-side psychological trade-offs. By detailing these historical limitations, Table 1 demonstrates why a dual PMT-TTF model—validated through an advanced machine learning framework—is required to uncover the non-linear behavioral criteria dictating how contemporary chronic patients cope with active cybersecurity risks.

## 3 Research model and hypotheses development

**Figure 1:**
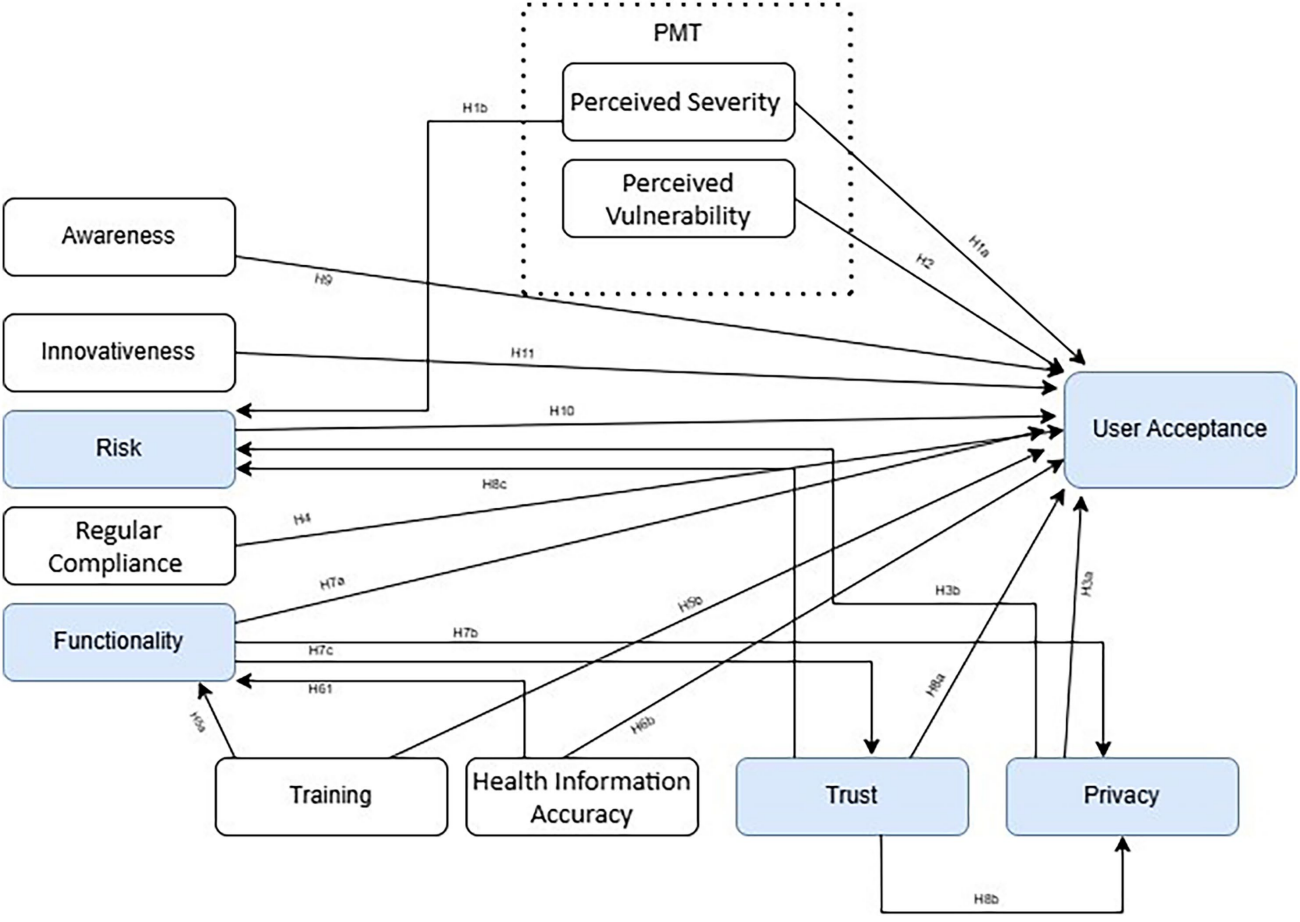
Research model. Inner model (Hypothesis). Colors indicate endogenous constructs.

In order to understand cybersecurity behavior and the intention to use the IoT in the healthcare context, the current study developed and applied the integrated model. The framework links constructs from PMT, TTF, and selected socio-technical factors through 19 explicit hypotheses, as shown in Figure 1.

### 3.1 Perceived severity (*PS*)

Perceived severity, derived from PMT, evaluates an individual’s cognitive assessment regarding the seriousness of a threat’s consequences (37). In clinical technology domains, when patients perceive their underlying medical condition as highly severe, their health threat appraisal increases, driving a willingness to adopt digital monitoring interventions (38). Empirical studies confirm that higher threat awareness among healthcare users positively correlates with their intention to adopt IoT systems to mitigate systemic health risks (39). Concurrently, within cyber-physical systems, a high perception of threat severity naturally amplifies a user’s sensitivity to structural vulnerabilities, thereby directly adjusting their baseline calculation of risk. Thus, we hypothesize:

*H1a*: Perceived severity has a direct positive effect on user acceptance.

*H1b*: Perceived severity has a direct positive effect on perceived risk.

### 3.2 Perceived vulnerability (*PV*)

Perceived vulnerability represents an individual’s subjective assessment of their susceptibility to a specific hazard or adverse event (24). Within digital healthcare literature, higher levels of perceived health vulnerability consistently act as a primary driver for technology adoption among geriatric and vulnerable demographics (19). When chronic patients believe they face an immediate risk of health destabilization, their intrinsic motivation to deploy defensive technologies (such as remote IoT tracking arrays) increases significantly to safeguard their well-being (37)Therefore, we hypothesize:

*H2*: Perceived vulnerability has a direct positive effect on user acceptance.

### 3.3 Privacy (*PRV*)

Privacy refers to the degree of sovereign control an individual maintains over the collection, dissemination, and handling of their personal health information (40). In remote health tracking networks, maintaining confidentiality is essential; users must trust that unauthorized actors cannot access sensitive telemetry data (41)If patients suspect that their digital health infrastructure exposes their diagnostic records to external actors, they experience acute psychological friction, resulting in technology rejection (37). Conversely, when strong data confidentiality is guaranteed, the baseline perception of systemic risk decreases. While some localized studies suggest that regulatory oversights can decouple risk from privacy perceptions (42)the broader literature indicates a direct causal link. Thus, we hypothesize:

*H3a*: Privacy has a direct positive effect on user acceptance.

*H3b*: Privacy has a direct negative effect on perceived risk.

### 3.4 Regular compliance (*RC*)

Regular compliance can be defined as the existence of healthcare rules and regulations set by the government to promote technology adoption and user compliance with these rules and regulations (43). A study indicated that an appropriate regulatory framework would favorably influence the healthcare sector (44). Hence, the absence of regulatory laws and regulations would lead to inadequate standards and flawed action instructions. In contrast, Cleveland and Haddara (45) reported that patients and healthcare workers sought to utilize the IoT for improved diabetes management via data sharing, but data ownership and privacy regulation issues created confusion and hindered technology adoption. Thus, we hypothesize:

*H4*: Regular compliance has a direct positive effect on user acceptance.

### 3.5 Training (*TRA*)

Training encompasses the formal mechanisms and educational resources provided to users to ensure they possess the necessary skills to operate a technology efficiently (46), when chronic patients receive comprehensive, structured guidance on how to navigate IoT devices, they optimize their usage patterns, unlocking the full functional capabilities of the technology.

Empirical research shows that adequate instructional onboarding reduces user frustration and directly shapes a positive attitude toward technology integration (47). By bridging the digital literacy gap, training enhances both functional execution and user acceptance. Thus, we hypothesize:

*H5a*: Training has a direct positive effect on functionality.

*H5b*: Training has a direct positive effect on user acceptance.

### 3.6 Health information accuracy (*HIA*)

Accuracy of health information refers to the degree of credibility and reliability of information related to health status provided by an IoT device (19). The IoT enhances patient-centered healthcare by gathering meaningful and precise health data appropriate to individuals’ comprehension and capabilities (48). A study (19) indicated that individuals were willing to check their health data when they considered the information from the IoT device to be trustworthy, consequently, classifying accuracy as a potential facilitator for using IoT devices. Accordingly, the current study hypothesizes that:

*H6a*: Health information accuracy has a direct positive effect on functionality.

*H6b*: Health information accuracy has a direct positive effect on user acceptance.

### 3.7 Functionality (*FN*)

Functionality represents the structural capacity of a technological system to execute specialized tasks reliably and consistently according to design specifications (41). When a digital platform exhibits high operational functionality, it provides a stable environment that directly reinforces user perceptions of privacy and systemic security (41). In medical contexts, robust device functionality assures patients that the tool will reliably perform its core clinical tracking tasks without system failures. This reliability helps build user trust and directly increases long-term acceptance (41). Thus, we hypothesize:

*H7a*: Functionality has a direct positive effect on user acceptance.

*H7b*: Functionality has a direct positive effect on privacy.

*H7c*: Functionality has a direct positive effect on trust.

### 3.8 Trust (*T*)

Trust is defined as the psychological state wherein a patient maintains absolute confidence in the reliability, safety, and integrity of an automated digital health service (49). According to Social Exchange Theory (SET), an individual’s willingness to perform a behavior depends heavily on trust, which serves as a cognitive mechanism to reduce uncertainty (49). In digital health networks, where personal metrics are vulnerable to intercept threats, establishing trust is crucial for user validation (18). Empirical studies confirm that long-term technology adoption is unsustainable without reliable privacy assurances that foster user trust (50). Consequently, trust serves as a primary psychological filter that neutralizes risk and accelerates technology acceptance (42). Therefore, we hypothesize:

*H8a*: Trust has a direct positive effect on user acceptance.

*H8b*: Trust has a direct positive effect on privacy.

*H8c*: Trust has a direct negative effect on risk.

### 3.9 Awareness (*AW*)

Awareness refers to how individuals think the IoT can improve the current state of disease control and monitoring (51). Awareness has been referenced several times in previous literature, whether regarding the awareness of diseases themselves or the IoT devices that monitor these diseases (51). Moreover, the study in (52) divided potential consumers into aware and unaware groups, finding that consumers’ awareness of the device’s properties was likely to influence their intention to adopt that device. The current study focuses on examining patient awareness of IoT devices, including their benefits and downsides. Subsequently, this hypothesis was formulated:

*H9*: Awareness has a direct positive effect on user acceptance.

### 3.10 Risk (*R*)

Perceived Risk in this study is anchored within Perceived Risk Theory (PRT) (53) and operationalized specifically as a two-dimensional construct matching the healthcare cyber-physical environment. First, Privacy/Data Risk, which is the subjective probability that a patient’s sensitive clinical telemetry (e.g., real-time cardiac waveforms or HIV status records) will be intercepted, leaked, or maliciously commercialized due to weak infrastructure (54). Second, Physical Safety Risk which is he terrifyingly concrete probability that a security breach will result in direct physical injury or death—such as an attacker hijacking an IoT insulin pump to deliver a fatal bolus, or jamming a pacemaker signal (55). Under Social Exchange Theory, trust acts as a critical information filter that directly reduces a user’s calculation of perceived risk (49). In a complex healthcare IoT environment, patients lack the technical expertise to evaluate cryptographic strengths or firmware security patches. This creates cognitive vulnerability. Trust serves as a psychological shortcut. When a patient develops trust in an IoT service (driven by institutional reputation or device functionality), they project a positive expectation onto the technology’s future performance (42). This projection cognitively lowers their assessment of threat probability. Mechanistically, trust shifts the patient’s focus away from catastrophic failure modes and onto the expected clinical benefits of the system. This cognitive realignment scales down the perceived risk curve, effectively neutralizing adoption paralysis. Consequently, users of IoT devices with a high-risk tendency are more resistant to adopting an IoT device, leading to the following hypothesis:

*H10*: Risk has a direct negative effect on user acceptance.

### 3.11 Innovativeness (*IN*)

Personal innovativeness evaluates an individual’s intrinsic willingness to experiment with, adopt, and incorporate novel technological paradigms ahead of their peers (56). Highly innovative individuals typically demonstrate a higher tolerance for technical ambiguity and navigate digital systems with lower risk aversion (57). In mobile health contexts, empirical studies confirm that high personal innovativeness positively shifts behavioral intentions toward adopting wearable tech, helping users overcome initial usage anxieties (52, 58). Thus, we hypothesize:

*H11*: Innovativeness has a direct positive effect on user acceptance.

### 3.12 User acceptance (user behavioral intention) (*UA*)

The elements that motivate IoT device adoption (59) User satisfaction, or user acceptance, has been documented at several levels in previous literature. For example, in (50) it was noted that higher satisfaction levels among nurses and physicians were associated with several aspects, including usefulness, dependability, and assistance offered when dealing with issues. Furthermore, some felt comfortable stating that examining the human aspects was essential for using IoT technology in hospitals and facilitating the interchange of health information (60). This illustrates the significance of this factor and its important role as an independent variable in the current study.

## 4 Research methodology

### 4.1 Research instrument development and validation

A quantitative survey design was employed to capture the empirical relationships within the proposed framework. The measurement items were adapted from established information systems (IS) and healthcare technology literature, with minor contextual modifications. Each latent construct was operationalized using two to five items (see Appendix Table A1), evaluated on a seven-point Likert scale ranging from “strongly disagree” (1) to “strongly agree” (7) (61). To accommodate the target demographic, the instrument was also translated into Arabic using a rigorous forward-and-backward translation protocol and hosted on a bilingual digital platform.

Content validity and structural clarity were established through an expert review panel (62). The panel comprised two doctoral researchers in cybersecurity/IS, two professors specializing in Information Systems, and a medical consultant specializing in chronic disease management. The experts evaluated the questionnaire’s formatting, semantic clarity, operational usability, and completion velocity. Based on their evaluations of structure, usability, and translation accuracy, minor modifications were made.

Beyond qualitative expert cross-checking, we have explicitly detailed that the scale underwent a formal pilot test (*n* = 50) to optimize phrasing and readability. Its statistical validity and internal reliability were further evaluated during the results stage using standardized indicator factor loadings, Cronbach’s alpha, Composite Reliability (CR), and Average Variance Extracted (AVE) parameters, all of which met strict empirical thresholds.

### 4.2 Sampling and data collection

The target population comprised chronic disease patients in the Kingdom of Saudi Arabia who were familiar of Internet of Things (IoT) health monitoring systems. This setting is particularly relevant given Saudi Arabia’s rapid health-tech transformation and infrastructural progress in digital healthcare technology. Following formal institutional approval from the Qassim Health Cluster, a dual-channel sampling strategy was executed. First, digital distribution across King Saud Hospital and its affiliated primary healthcare centers. Second, in-person recruitment was conducted at localized diabetes clinics and patient support groups, leveraging snowball sampling to maximize reach. A total of 850 individuals within the targeted chronic patient demographics were originally contacted across medical networks. Of these, 142 individuals actively declined to participate, and 105 surveys were discarded during data cleaning due to missing values or pattern responses. This yielded a final usable sample size of *n* = 603 fully completed and valid observations (representing a net functional response rate of 70.9%). While the minimum sample threshold was initially established at 300 participants based on the conservative “10 times” structural rule for Partial Least Squares (PLS) path estimation (63). The final data collection phase yielded a highly robust sample of *n* = 603 valid responses, substantially enhancing statistical power and predictive generalizability.

### 4.3 Analytical approach: hybrid PLS-SEM and ANN

To achieve both causal explanation and robust predictive validation, this study adopted an advanced two-stage hybrid analytical design combining Partial Least Squares Structural Equation Modeling (PLS-SEM) and Artificial Neural Networks (ANN) (64, 65). The first stage is PLS-SEM. It is executed using SmartPLS software following a standard two-step estimation approach (66). The first phase evaluates the measurement (outer) model to verify internal consistency reliability, convergent validity, and discriminant validity. The second phase tests the structural (inner) model to evaluate linear path coefficients to test hypothesized relationships between latent constructs. PLS-SEM was chosen due to its high flexibility regarding non-normal distribution parameters and its superior performance in mapping highly complex multi-construct frameworks (67). The second stage is ANN. It is implemented as a secondary, machine-learning tool to process the model’s variables. This multi-analytical approach overcomes the inherent limitations of standard linear regression models by capturing deep, non-linear, and non-compensatory human behavioral patterns, yielding highly accurate normalized importance rankings (64).

## 5 Results

In review, the findings indicate higher participation in the study by female participants (61%, *n* = 368) compared to male participants (39%, *n* = 235). This imbalance may reflect gender-specific health-seeking behaviors or engagement with chronic disease management. Moreover, most participants were concentrated in the younger age groups (18–40 years) (i.e., 18–30 years, *n* = 180) participants [29.9%]; 30–40 years, *n* = 184 participants [30.5%], accounting for over 60% of the sample, with 18.7% (*n* = 113 participants) aged 40–50 years This suggests that younger patients may be more inclined to adopt technology and IoT devices for health monitoring. However, the presence of participants aged over 50 (20.9%) also indicates potential interest or a need for IoT solutions among older populations.

### 5.1 Measurement model evaluation

Following the guidelines established by Hair et al. (66), the reflective measurement model was evaluated using four criteria: indicator reliability, internal consistency reliability, convergent validity, and discriminant validity.

#### 5.1.1 Indicator reliability

Indicator reliability assesses the extent to which an item’s variance is captured by its underlying construct. A standardized factor loading threshold of 0.708 or higher is required, confirming that the construct explains at least 50% of the indicator’s variance (66). During initial model estimation, three items—RC1 and RC5 from Regulatory Compliance, and PRV4 from Privacy—exhibited inadequate factor loadings and were systematically removed to optimize model fit. Following this refinement, all remaining standardized factor loadings ranged from 0.798 to 0.928 (see Table 2), demonstrating robust indicator reliability.

**Table 2:** Reliability and validity of constructs.

Construct	Items	Outer loadings	Cronbach’s alpha (*α*)	Composite reliability (*rho_A*)	Composite reliability (*rho_c*)	Average variance extracted (AVE)
Awareness	*AW1*	0.807	0.822	0.852	0.893	0.736
	*AW2*	0.867				
	*AW3*	0.898				
Functionality	*FN1*	0.928	0.911	0.912	0.944	0.850
	*FN2*	0.926				
	*FN3*	0.911				
Health information accuracy	*HIA1*	0.949	0.889	0.889	0.947	0.900
	*HIA2*	0.948				
Innovativeness	*IN1*	0.814	0.775	0.777	0.869	0.689
	*IN2*	0.826				
	*IN3*	0.850				
Privacy	*PRV1*	0.878	0.882	0.882	0.927	0.809
	*PRV2*	0.921				
	*PRV3*	0.899				
Perceived severity	*PS1*	0.872	0.806	0.818	0.885	0.720
	*PS2*	0.874				
	*PS3*	0.798				
Perceived vulnerability	*PV1*	0.901	0.902	0.902	0.938	0.836
	*PV2*	0.923				
	*PV3*	0.918				
Risk	*R1*	0.850	0.853	0.855	0.901	0.694
	*R2*	0.852				
	*R3*	0.826				
	*R4*	0.803				
Regular compliance	*RC2*	0.891	0.842	0.843	0.905	0.760
	*RC3*	0.870				
	*RC4*	0.854				
Trust	*T1*	0.868	0.916	0.917	0.941	0.799
	*T2*	0.903				
	*T3*	0.898				
	*T4*	0.907				
Training	*TRA1*	0.916	0.899	0.899	0.937	0.832
	*TRA2*	0.917				
	*TRA3*	0.902				
User acceptance	*UA1*	0.862	0.844	0.846	0.906	0.762
	*UA2*	0.861				
	*UA3*	0.896				

#### 5.1.2 Internal consistency reliability

Internal consistency reliability ensures that the multi-item scales consistently measure their intended constructs. This was evaluated using three distinct metrics: Cronbach’s Alpha, Composite Reliability (rho_A), and Composite Reliability (rho_c). Grounded in suggested thresholds, a value of 0.70 serves as the minimum acceptable baseline for established research, while values exceeding 0.95 indicate item redundancy (68) All Cronbach’s Alpha values surpassed 0.70 without exceeding the upper limit. Similarly, the constructs exhibited rho_c values between 0.869 and 0.947, comfortably exceeding the standard 0.70 to 0.95 benchmark (69). Finally, all rho_A coefficients cleared the recommended 0.70 threshold, providing a robust intermediate balance between Cronbach’s Alpha and rho_c (70).

#### 5.1.3 Convergent validity

Convergent validity evaluates the degree to which indicators mathematically converge to represent a single latent variable. This parameter is operationalized via the Average Variance Extracted (AVE), where a minimum threshold of 0.50 is required to demonstrate that a construct accounts for the majority of its indicators’ variance (70). As documented in Table 2, all computed AVE values cleared the 0.50 baseline, confirming satisfactory convergent validity across the framework.

#### 5.1.4 Discriminant validity

Discriminant validity ensures that each latent construct within the model is empirically independent. This was validated using cross-loadings, the Fornell–Larcker criterion, and the Heterotrait–Monotrait (HTMT) ratio. First, cross-loading evaluation confirmed that every measurement indicator exhibited a higher standardized factor loading on its designated target construct than on any other latent construct (see Appendix Table A2) (69). Second, according to the Fornell–Larcker criterion, the square roots of the AVEs (displayed in bold along the diagonal in Table 3) were uniformly greater than the corresponding off-diagonal construct correlations, validating the distinctiveness of each variable (69). Finally, the HTMT ratio was applied as a sensitive metric for uncovering hidden discriminant issues (69). Adhering to strict methodological guidelines, an HTMT ratio below 0.85 or 0.90 is required depending on construct proximity (71). As shown in Table 4, all generated HTMT ratios fell comfortably below the conservative 0.90 ceiling, providing final empirical confirmation of discriminant validity.

**Table 3:** Fornell–Larcker criterion.

Factors name	*AW*	*FN*	*HIA*	*IN*	*PRV*	*PS*	*PV*	*R*	*RC*	*T*	*TRA*	*UA*
*AW*	**0.858**											
*FN*	0.282	**0.922**										
*HIA*	0.321	0.528	**0.949**									
*IN*	0.422	0.217	0.268	**0.830**								
*PRV*	0.231	0.688	0.367	0.165	**0.900**							
*PS*	0.344	0.266	0.276	0.304	0.290	**0.849**						
*PV*	0.339	0.231	0.274	0.264	0.248	0.368	**0.914**					
*R*	0.383	0.540	0.275	0.378	0.623	0.361	0.382	**0.833**				
*RC*	0.401	0.317	0.344	0.236	0.285	0.381	0.442	0.358	**0.872**			
*T*	0.302	0.733	0.438	0.258	0.671	0.326	0.332	0.629	0.313	**0.894**		
*TRA*	0.297	0.609	0.645	0.251	0.461	0.336	0.343	0.395	0.378	0.507	**0.912**	
*UA*	0.511	0.303	0.311	0.455	0.349	0.538	0.504	0.514	0.529	0.388	0.343	**0.873**

The square roots of the AVEs (displayed in bold along the diagonal).

**Table 4:** Heterotrait–monotrait (HTMT) ratio.

Factors name	*AW*	*FN*	*HIA*	*IN*	*PRV*	*PS*	*PV*	*R*	*RC*	*T*	*TRA*	*UA*
*AW*												
*FN*	0.324											
*HIA*	0.377	0.587										
*IN*	0.522	0.257	0.325									
*PRV*	0.266	0.767	0.414	0.199								
*PS*	0.412	0.309	0.324	0.383	0.342							
*PV*	0.390	0.254	0.306	0.313	0.277	0.428						
*R*	0.445	0.611	0.314	0.461	0.718	0.433	0.435					
*RC*	0.479	0.361	0.398	0.289	0.327	0.460	0.507	0.423				
*T*	0.342	0.802	0.485	0.303	0.745	0.377	0.366	0.711	0.355			
*TRA*	0.340	0.673	0.721	0.300	0.517	0.392	0.381	0.450	0.434	0.557		
*UA*	0.601	0.347	0.359	0.560	0.405	0.646	0.579	0.605	0.627			

### 5.2 Structural model evaluation

**Figure 2:**
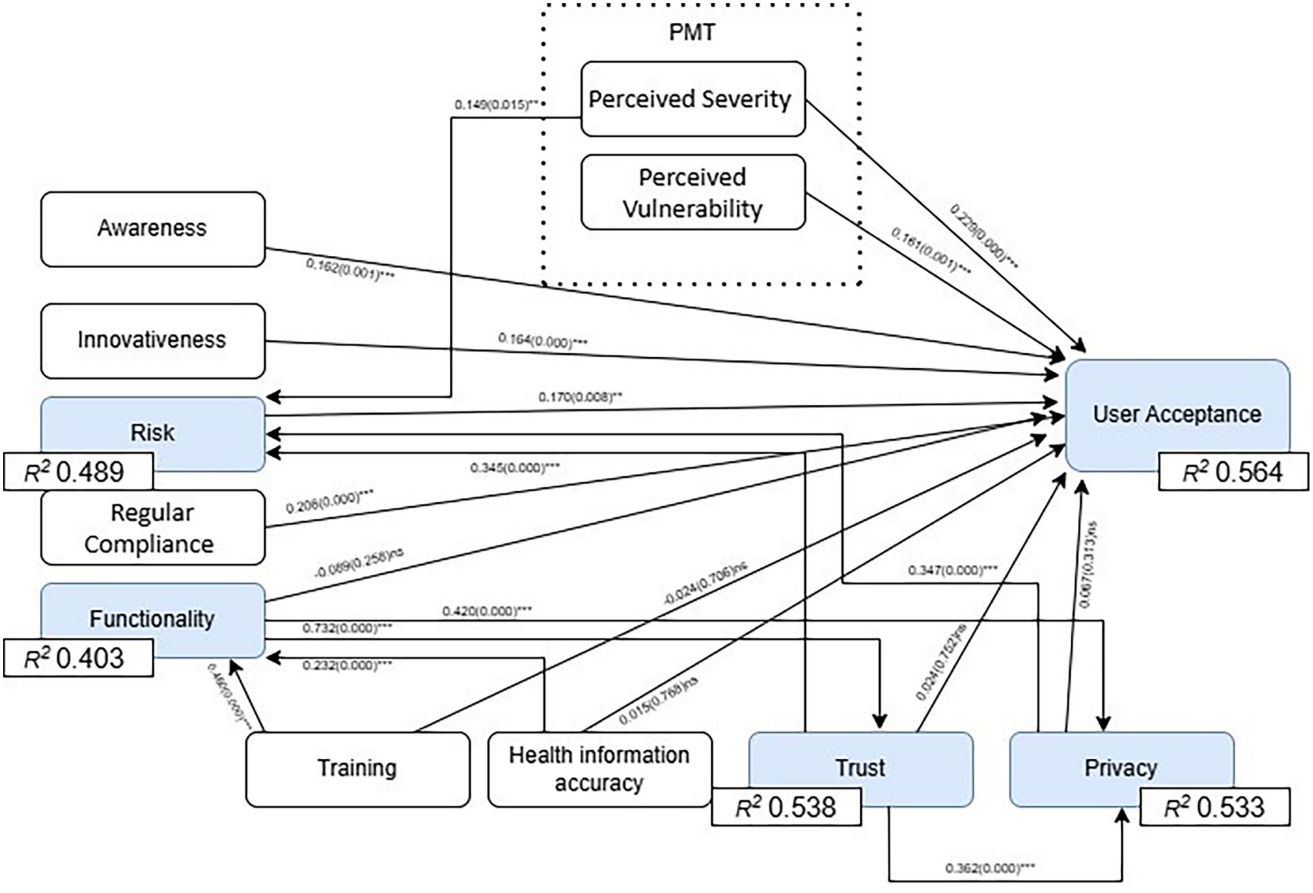
Structural model results. Inner model (path coefficients and *p*-values); ns, non-significant; ∗*p* < 0.05; ∗∗*p* < 0.01; ∗∗∗*p* < 0.001. Colors indicate endogenous constructs.

This study employed the following standard metrics to assess the structural model: evaluation of collinearity, followed by the assessment of path coefficients, then coefficients of determination (*R*^2^ value), effect size (*f*^2^ value), and lastly, predictive significance (*Q*^2^). Figure 2 presents the results of the structural model evaluation.

Prior to evaluating the structural model’s path configurations, a rigorous multi-stage collinearity check was performed to confirm structural stability. While calculating Variance Inflation Factors (VIF) is a standard mechanism in structural equations, over-reliance on VIF metrics alone can overlook localized indicator-level overlap within multi-stage predictive algorithms. Therefore, this study cross-checked potential collinearity through a two-pronged approach: assessing internal inner VIF vectors alongside an item-by-item cross-loadings matrix. All inner VIF parameters fell significantly below the conservative threshold of 3.3, and indicator loadings paired cleanly with their respective constructs, confirming that the structural relationships are entirely free from multi-collinearity inflation distortions before applying the non-linear machine learning parameters.

#### 5.2.1 Assessment size and significance of path coefficients

**Figure 3:**
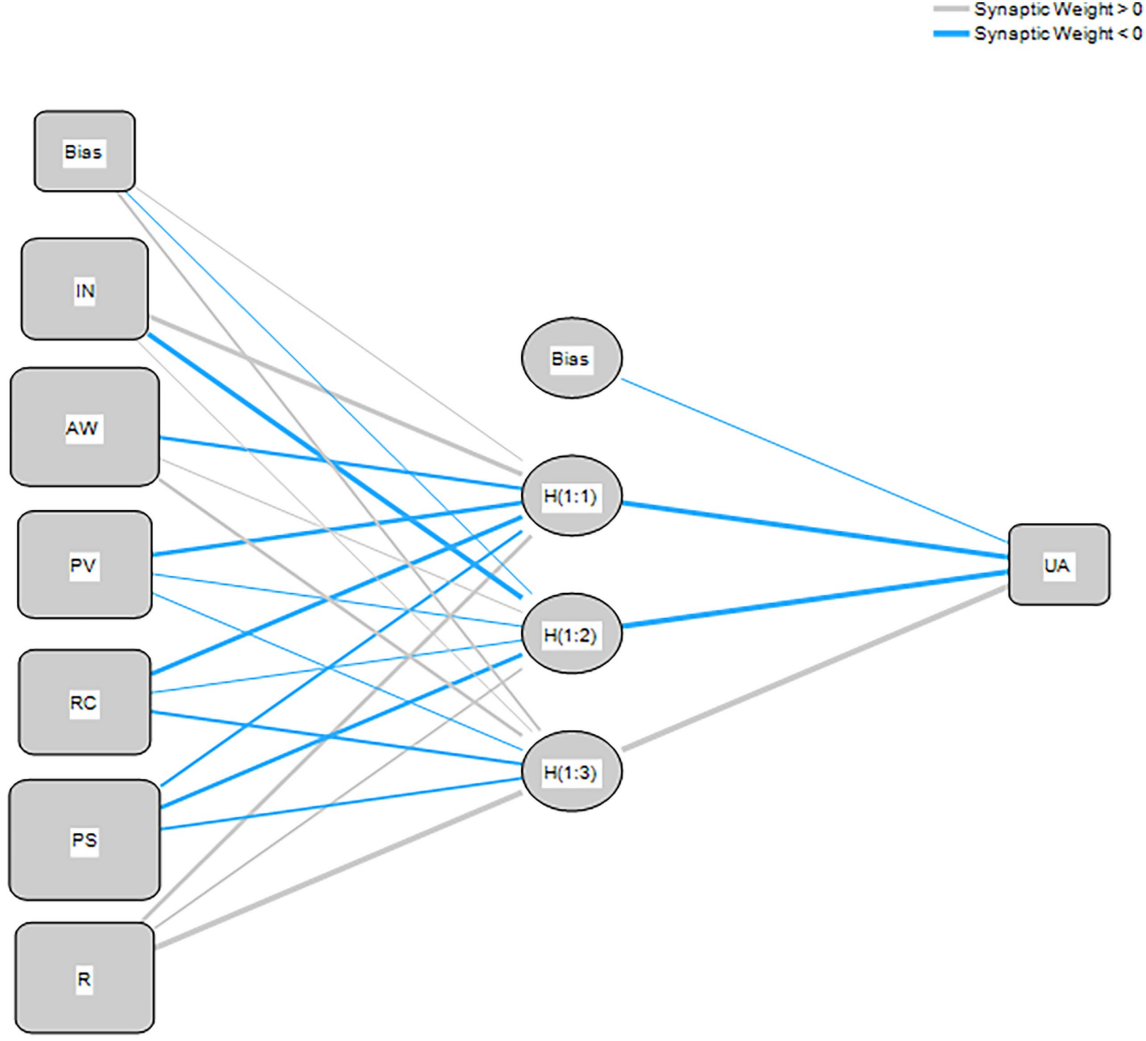
Artificial neural network (ANN) model. Hidden layer activation function: hyperbolic tangent; input neurons: IN, innovativeness; AW, awareness; PV, perceived severity; RC, regular compliance; PS, perceived vulnerability; R, risk; UA, user acceptance.

In terms of significance, prior studies have often recommended that this be evaluated using bootstrapping to provide standard errors, *t*-statistic values, and *p*-values (72). The bootstrapping procedure produces *p*-values and *t*-statistic values to examine the statistical significance and relevance (i.e., the size) of the path coefficient. Nunnally and Bernstein (73) specified the following standard measures: *t*-values above 1.96 (two-tailed) for a 5% significance level; exceeding 2.68 for a 1% significance level; and above 3.29 for a 0.1% significance threshold. Finally, based on a common recommendation by Streukens and Leroi-Werelds (74), the current study used a large number of bootstrap subsamples (i.e., 10,000) for the final results to ensure stability. The path analysis and hypotheses’ testing results are presented in Figure 3 and summarized in Table 5. As shown, of the 19 directly hypothesized relationships in the study’s research model, the findings revealed that the following five were non-significant: (*FN* ➔ *UA*), (*HIA* ➔ *UA*), (*PRV* ➔ *UA*), (*T* ➔ *UA*), and (*TRA* ➔ *UA*). In addition, the results showed the following 12 positive, highly significant relationships: (*HIA* ➔ *FN*), (*TRA* ➔ *FN*), (*PRV* ➔ *R*), (*T* ➔ *R*), (*FN* ➔ *PRV*), (*T* ➔ *PRV*), (*FN* ➔ *T*), (*AW* ➔ *UA*), (*IN* ➔ *UA*), (*PS* ➔ *UA*), (*PV* ➔ *UA*), and (*RC* ➔ *UA*). Finally, the following two relationships were found to be positive and significant: (*PS* ➔ *R*) and (*R* ➔ *UA*).

**Table 5:** Results of hypotheses’ testing.

Hypotheses	Association	Sample mean (M) (*β*)	Standard deviation (SD)	*t-* statistics	*p-*values	Supported
1a	*PS ➔ UA*	0.229	0.051	4.575	0.000***	YES
1b	*PS ➔ R*	0.149	0.060	2.439	0.015**	YES
2	*PV ➔ UA*	0.161	0.047	3.448	0.001***	YES
3a	*PRV ➔ UA*	0.067	0.063	1.010	0.313	NO
3b	*PRV ➔ R*	0.347	0.070	4.931	0.000***	YES
4	*RC ➔ UA*	0.206	0.046	4.549	0.000***	YES
5a	*TRA ➔ FN*	0.460	0.052	8.870	0.000***	YES
5b	*TRA ➔ UA*	−0.024	0.062	0.377	0.706	NO
6a	*HIA ➔ FN*	0.232	0.066	3.517	0.000***	YES
6b	*HIA ➔ UA*	0.015	0.055	0.296	0.768	NO
7a	*FN ➔ UA*	−0.089	0.081	1.130	0.258	NO
7b	*FN ➔ PRV*	0.420	0.083	5.098	0.000***	YES
7c	*FN ➔ T*	0.732	0.042	17.615	0.000***	YES
8a	*T ➔ UA*	0.024	0.078	0.317	0.752	NO
8b	*T ➔ PRV*	0.362	0.077	4.663	0.000***	YES
8c	*T ➔ R*	0.345	0.067	5.198	0.000***	YES
9	*AW ➔ UA*	0.162	0.048	3.423	0.001***	YES
10	*R ➔ UA*	0.170	0.062	2.659	0.008**	YES
11	*IN ➔ UA*	0.164	0.043	3.813	0.000***	YES

***p < 0.001 highly significant, **p < 0.01 significant, *p < 0.05 marginally significant.

#### 5.2.2 Assessment of coefficients of determination (*R*^2^ values)

The *R*^2^ value indicates the variance explained in each endogenous construct and serves as a metric for the model’s explanatory power, as concluded by Shmueli and Koppius (75), and also known as in-sample predictive power. The *R*^2^ values of 0.75, 0.50, and 0.25 are regarded as substantial, moderate, and weak, respectively, indicating that the model’s explanatory power enhances as *R*^2^ values rise (76). In the current study, as shown in Table 6, the total variance explained by the research model, as shown by its *R*^2^ values, was 0.403 for *FN* attributed to *HIA* and *TRA*, and 0.489 for *R* attributed to *PRV*, *PS*, and *T*, which is considered to be weak predictive accuracy; and 0.533 for *PRV* attributed to *FN* and *T*, 0.538 for *T* attributed to *FN*, and 0.564 for *UA* attributed to *AW, FN, HIA, IN, PRV, PS, PV, R, RC, T*, and *TRA* which is considered to be moderate overall predictive accuracy. The variation for *FN* was 40.3, 53.3% for *PRV*, 48.9% for *R*, 53.8% for T, and, lastly, 56.4% for *UA*, indicating a moderate degree of predictive accuracy.

**Table 6:** Coefficient of determination (R^2^), predictive relevance (Q^2^), and effect size (f^2^).

Endogenous (dependent) variables	*R* ^2^	*Q* ^2^	Exogenous (independent) variables	*f* ^2^	Effect size
*FN*	0.403	0.389	*HIA*	0.053	Small Effect
			*TRA*	0.207	Medium Effect
*PRV*	0.533	0.209	*FN*	0.178	Medium Effect
			*T*	0.128	Small Effect
*R*	0.489	0.183	*PRV*	0.128	Small Effect
			*PS*	0.037	Small Effect
			*T*	0.127	Small Effect
*T*	0.538	0.265	*FN*	1.164	Large Effect
*UA*	0.564	0.511	*AW*	0.041	Small Effect
			*FN*	0.006	No Effect
			*HIA*	0.000	No Effect
			*IN*	0.046	Small Effect
			*PRV*	0.004	No Effect
			*PS*	0.090	Small Effect
			*PV*	0.042	Small Effect
			*R*	0.028	Small Effect
			*RC*	0.068	Small Effect
			*T*	0.000	No Effect
			*TRA*	0.001	No Effect

#### 5.2.3 Assessment of effect size (f^2^) value

The practical relevance of each construct was evaluated using Cohen’s (77) effect size (*f*^2^) thresholds, where 0.02, 0.15, and 0.35 represent small, medium, and large effects, respectively. As detailed in Table 6, FN exerted a small effect on HIA and a medium effect on TRA, while PRV showed a medium effect on FN and a small effect on T. Perceived Risk (R) had small effects on PRV and T, but large effects on FN and T. Crucially, regarding the ultimate dependent variable, User Acceptance (UA), the exogenous predictors AW, IN, PS, PV, R, and RC generated small effect sizes (*f*^2^ > 0.02), while FN, HIA, PRV, T, and TRA produced no practical effect (*f*^2^ < 0.02). They indicate that while threat appraisals and risk factors are statistically validated components of the model, they offer limited practical leverage over final adoption decisions.

#### 5.2.4 Assessment of predictive relevance (*Q*^2^ value)

Predictive relevance (*Q*^2^) assesses the model’s out-of-sample predictive capacity, with a value greater than 0 indicating that the latent construct values are accurately reconstructed (78). Computed via the PLSpredict method in SmartPLS, the metric was generated using a 10-fold cross-validation configuration (K = 10, *r* = 10) adhering to rigorous validation standards (79). Thresholds of 0.02, 0.15, and 0.35 signify weak, moderate, and strong predictive relevance, respectively (80). The empirical outputs confirmed strong out-of-sample predictive relevance across the framework, as all endogenous *Q*^2^ values were comfortably above zero. Specifically, Perceived Risk (*Q*^2^ = 0.183) and Privacy (*Q*^2^ = 0.209) exhibited moderate-to-strong predictive relevance. Meanwhile, Functionality (*Q*^2^ = 0.389), Trust (*Q*^2^ = 0.265), and the ultimate focal construct, User Acceptance (*Q*^2^ = 0.511), all demonstrated exceptionally strong predictive relevance, confirming the high robustness of the integrated model.

### 5.3 Assessment of predictive power employing PLSpredict

To rigorously evaluate the out-of-sample predictive power of the structural framework, this study executed the PLSpredict algorithm following the methodology established by Shmueli et al. (79)This procedure involves partitioning the dataset to train the model parameter estimations on an analysis sample and systematically evaluating its predictive execution on a holdout sample (79)To maximize statistical stability and minimize partitioning bias, a 10-fold cross-validation procedure (K = 10) with 10 conceptual repetitions (*r* = 10) was implemented, fully satisfying minimum sample criteria thresholds.

A critical requirement of the PLSpredict protocol is comparing the prediction error metrics of the structural model against a naive Linear Model (LM) benchmark. This naive LM benchmark is constructed automatically by running an Ordinary Least Squares (OLS) regression for each individual indicator of the endogenous constructs against all exogenous indicators in the model. Because this baseline linear model completely ignores the hypothesized structural path configurations and structural groupings, it serves as a strict, non-structural baseline.

Following Shmueli et al.’s (79)guidelines, the model’s out-of-sample predictive power is determined by comparing the Root Mean Square Error (RMSE) or Mean Absolute Error (MAE) values of the PLS-SEM factors against this naive LM baseline. Visual inspection of our endogenous construct error distributions revealed non-symmetric, skewed patterns; thus, the MAE metric was selected as the mathematically appropriate indicator for asymmetric error distributions, as it does not disproportionately penalize larger outlier errors.

The predictive evaluation rules are strictly structured: if all PLS-SEM indicators yield lower error metrics than the naive LM baseline, high predictive power is established; a majority or equal distribution signifies medium predictive power; whereas a minority signifies low predictive capabilities (79). As detailed in Table 7, all indicators generated positive *Q*^2^ values, confirming genuine predictive relevance. Furthermore, a critical comparison demonstrated that a majority of the indicators—specifically nine out of 17 achieved lower MAE scores in the PLS-SEM analysis compared to the naive OLS linear regression benchmarks. This empirical distribution rigorously confirms that the structural model developed in this study possesses a robust medium predictive capability, outperforming a standard, unstructured linear baseline.

**Table 7:** PLSpredict results.

Items	Q^2^ predict	PLS-SEM_RMSE	PLS-SEM_MAE	LM_RMSE	LM_MAE	PLS-SEM RMSE_LM_ RMSE	PLS-SEM MAE_ LM_MAE
*FN1*	0.319	0.882	0.634	0.905	0.648	−0.023	−0.014
*FN2*	0.361	0.854	0.629	0.884	0.641	−0.03	−0.012
*FN3*	0.31	0.91	0.673	0.941	0.69	−0.031	−0.017
*T1*	0.211	0.963	0.721	0.976	0.728	−0.013	−0.007
*T2*	0.196	0.98	0.741	0.997	0.735	−0.017	0.006
*T3*	0.231	0.988	0.743	0.994	0.743	−0.006	0
*T4*	0.206	0.999	0.767	1.024	0.766	−0.025	0.001
*PRV1*	0.143	1.148	0.891	1.185	0.92	−0.037	−0.029
*PRV2*	0.176	1.048	0.803	1.075	0.814	−0.027	−0.011
*PRV3*	0.185	1.118	0.877	1.121	0.852	−0.003	0.025
*R1*	0.123	1.129	0.907	1.1	0.856	0.029	0.051
*R2*	0.154	1.132	0.896	1.122	0.853	0.01	0.043
*R3*	0.111	1.129	0.882	1.11	0.856	0.019	0.026
*R4*	0.118	1.17	0.938	1.159	0.912	0.011	0.026
*UA1*	0.397	0.768	0.621	0.791	0.643	−0.023	−0.022
*UA2*	0.333	0.78	0.629	0.793	0.638	−0.013	−0.009
*UA3*	0.422	0.762	0.624	0.777	0.636	−0.015	−0.012

### 5.4 Artificial neural network (ANN)

To capture complex, non-linear interactions and ascertain the precise relative importance of the exogenous factors predicting User Acceptance (UA), this study implemented a Multi-Layer Perceptron (MLP) Artificial Neural Network (ANN) driven by a Feed-Forward Backpropagation (FFBP) learning algorithm. In accordance with established network design guidelines (81). The structural topology consists of three layers as shown in Figure 3. An input layer containing the six statistically validated exogenous constructs from the PLS-SEM phase (Regulatory Compliance, Perceived Risk, Perceived Severity, Awareness, Perceived Vulnerability, and Innovativeness), an hidden layer for intermediate computational mapping, and an output layer housing the target endogenous variable (User Acceptance).

To ensure optimization and transparency, the structural hyperparameters of the network were explicitly defined. The hidden layer utilized a Hyperbolic Tangent (tanh) activation function to process incoming non-linear signals, while the output layer employed an Identity activation function to systematically generate continuous scale scores. The optimal number of hidden neurons was autonomously determined using a trial-and-error algorithmic search within a single-layer topology. Network weights were updated via gradient descent, with the learning rate initialized at 0.01 and paired with a momentum coefficient of 0.4 to guarantee stable convergence and eliminate mathematical oscillations. To mitigate the risk of algorithmic overfitting, a robust 10-fold cross-validation procedure was executed, generating 10 distinct network models. In line with rigorous machine learning paradigms (82). the dataset was systematically partitioned, allocating 70% of the observations for network training (weight optimization) and 30% as a holdout testing sample for independent validation of predictive accuracy.

The predictive accuracy of the model was evaluated by calculating the Root Mean Square Error (RMSE) for both dataset fractions across all 10 folds. As presented in Table 8, the network yielded a mean RMSE of 0.4496 for the training phase and an exceptionally close mean RMSE of 0.4498 for the testing phase. The near-identical nature of these cross-validated error metrics rigorously demonstrates that the ANN model possesses robust predictive stability, is free from localized overfitting, and fits the underlying empirical data with high mathematical precision. After confirming predictive relevance, a multi-layer sensitivity analysis was conducted using the network’s non-zero synaptic weights to calculate the normalized relative importance of each input neuron (82).

**Table 8:** RMSE values for training and testing.

Network	Training		Testing		Root mean square error (RMSE)	
	SSE (sum of squared errors) training	Sample	SSE testing	Sample	Training	Testing
1	33.835	228	19.547	105	0.385	0.431
2	52.092	242	15.804	91	0.463	0.416
3	48.059	226	18.858	107	0.461	0.419
4	43.686	226	29.523	107	0.439	0.525
5	37.272	233	24.787	100	0.399	0.497
6	42.799	244	16.086	89	0.418	0.425
7	48.776	224	26.045	109	0.466	0.488
8	61.216	233	18.447	100	0.512	0.429
9	53.33	234	17.118	99	0.477	0.415
10	53.522	241	18.493	92	0.471	0.448
Mean	47.458	233.1	20.470	99.9	0.449	0.449
SD	7.806	6.862	4.419	6.862	0.036	0.037

As detailed in Table 9, Regulatory Compliance (RC) emerged as the primary predictor of patient User Acceptance (UA), achieving a normalized importance score of 100%. This was followed by Perceived Risk (R) at 91.20% and Perceived Severity (PS) at 79.80%. Finally, the secondary behavioral layers were occupied by Awareness (AW) at 75.20%, Perceived Vulnerability (PV) at 71.70%, and Personal Innovativeness (IN) at 57.40%.

**Table 9:** Sensitivity analysis.

Network	*IN*	*AW*	*PV*	*RC*	*PS*	*R*
1	0.089	0.171	0.136	0.277	0.127	0.2
2	0.082	0.177	0.143	0.227	0.236	0.136
3	0.17	0.091	0.123	0.221	0.132	0.264
4	0.113	0.19	0.13	0.227	0.138	0.202
5	0.139	0.149	0.15	0.162	0.191	0.209
6	0.122	0.081	0.218	0.233	0.201	0.146
7	0.132	0.129	0.154	0.214	0.182	0.189
8	0.087	0.236	0.107	0.173	0.129	0.268
9	0.145	0.185	0.159	0.154	0.19	0.167
10	0.129	0.174	0.19	0.216	0.153	0.138
Average importance	0.120	0.158	0.151	0.210	0.167	0.1919
Normalized importance (%)	57.40%	75.20%	71.70%	100	79.80%	91.20%

### 5.5 Common method bias (CMB)

To safeguard the structural integrity of the model, both procedural remedies and statistical controls were deployed to assess potential common method bias (CMB). Procedurally, scale items were thoroughly refined during a pre-test phase to maximize semantic clarity and eliminate ambiguity (83). Additionally, to disrupt automatic response patterns, the standard Likert-scale questions were occasionally interspersed with objective, multiple-choice operational items. Statistically, the data were subjected to Harman’s single-factor test using Exploratory Factor Analysis (EFA) in SPSS. The factor extraction revealed that the single largest factor accounted for only 33.446% of the total variance. Because this value falls substantially below the conventional 50% threshold (84), it confirms that no single dimension dominates the covariance matrix, indicating that CMB is not a concern to this study.

## 6 Discussion

### 6.1 Perceived severity (*PS*)

The empirical results demonstrate that Perceived Severity (PS) is a powerful driver of a patient’s behavioral intention to adopt health-related IoT devices. The direct structural path from PS to User Acceptance (UA) was highly significant (*β* == 0.229, *t*-value = 4.575, supporting H1a). Additionally, PS exhibited a statistically significant positive relationship with the internal assessment of health risks (*β* = 0.149, *t*-value = 2.439, supporting H1b). These findings are highly consistent with previous literature by (38), which established that an individual’s willingness to deploy health monitoring technologies is deeply tied to their cognitive evaluation of the severity of their medical condition. Similarly, Solangi et al. (39) noted that when patients perceive their underlying illness as severe, the heightened salience of health threats actively overrides technology friction, accelerating adoption. In the practical context of chronic disease management, this indicates that patients who view their diagnoses as high-stakes or potentially life-threatening exhibit a much higher readiness to integrate the Internet of Medical Things (IoMT) into their daily routines. They view continuous physiological tracking not as a technical burden, but as a critical clinical safety net, meaning that higher subjective health risks directly translate into proactive adoption behaviors.

#### 6.1.1 Perceived vulnerability (*PV*)

This study confirmed that Perceived Vulnerability (PV) exerts a highly significant positive effect on user acceptance of IoT devices (*β* = 0.161, *t*-value = 3.448, supporting H2). This finding strongly aligns with historical digital health adoption models detailing PV as a fundamental health threat appraisal, particularly demonstrating its predictive strength among geriatric cohorts (19) and across broader telemedicine applications (37). This relationship demonstrates that a chronic patient’s internal, somatic sense of physical vulnerability directly dictates their willingness to accept continuous monitoring systems. When an individual feels exposed to sudden, unpredictable health complications, their psychological barriers against technological intrusiveness are significantly lowered. This proves that emotional and body-conscious risk assessments are vital components of the technology acceptance calculus, requiring device developers to frame continuous monitoring as an empowering armor rather than an invasive surveillance tool.

#### 6.1.2 Privacy (PRV) and trust (T)

Counter to foundational Information Systems (IS) models that traditionally position privacy and trust as dominant, direct determinants of technology adoption (41, 49). This study revealed an unexpected structural phenomenon. Data privacy was found to have a non-significant direct impact on user acceptance (*β* = 0.067, *t*-value = 1.010, rejecting H3a), despite maintaining a strong, statistically significant negative relationship with perceived risk (*β* = 0.347, *t*-value = 4.931, supporting H3b). Parallelly, the direct structural path from Trust (T) to User Acceptance (UA) was completely non-significant (*β* = 0.024, *t*-value = 0.317, rejecting H8a).

To resolve these apparent empirical contradictions, this study introduces the Chronic Utility Paradox and the structural concept of Institutional Trust Subsumption. Chronic disease patients operate within a high-stakes, asymmetric risk landscape. They are forced to continuously balance an immediate, concrete physical threat (e.g., a fatal cardiac event or diabetic shock) against an abstract, deferred digital threat (e.g., data privacy compromise). Under the rules of bounded rationality, patients execute a critical life-preserving utility override: the immediate biological necessity of continuous physiological tracking completely eclipses individual data sovereignty anxieties. Furthermore, this non-significance is deeply driven by the localized institutional architecture of the Kingdom of Saudi Arabia. Under the digital transformation mandates of Saudi Vision 2030 and the Health Sector Transformation Program, public healthcare delivery and medical data exchanges have been highly centralized within state-administered digital networks, such as the Sehaty platform (9). Within this societal framework, there is an exceptionally high baseline of public confidence in state data governance, cyber-legislation, and public sector infrastructure. When an IoMT device is deployed within this ecosystem, a process of *macro-trust subsumption* occurs. Patients project their broad, institutional trust in the state directly onto the device itself, effectively treating data privacy and vendor integrity as an assumed, structural guarantee. Consequently, micro-level, individual calculations of vendor trust and device-specific privacy risks are neutralized, rendering them statistically non-significant in driving behavioral variance.

#### 6.1.3 Regulatory compliance (RC)

A highly significant positive relationship was established between Regulatory Compliance (RC) and User Acceptance (UA) (*β* = 0.206, *t*-value = 4.549, supporting H4). This finding strongly supports previous literature that highlights the critical nature of formal institutional compliance frameworks in highly sensitive, risk-averse sectors (44). In clinical settings, users experience an enhanced sense of structural security when they are assured that their devices strictly adhere to official national data protection protocols. Crucially, this finding directly challenges the conclusions of Cleveland and Haddara (45), which argued that rigid compliance regulations slow down or suppress IoT adoption due to increased user onboarding friction. In our target demographic, official regulatory compliance does not act as a procedural burden; instead, it serves as the primary psychological anchor of safety. Because patients operate under a centralized institutional trust umbrella, official regulatory endorsement acts as the definitive validation that allows them to bypass individual risk calculations and confidently adopt the technology.

#### 6.1.4 Training (TRA) and functionality (FN)

The empirical data revealed a highly significant positive relationship between Training (TRA) and device Functionality (FN) (*β* = 0.460, *t*-value = 8.870, supporting H5a), alongside a strong influence of Health Information Accuracy (HIA) on Functionality (β = 0.232, *t*-value = 3.517, supporting H6a). These results match Behavioral Reasoning Theory (BRT) and traditional system quality frameworks (46, 48). proving that extensive user training and high data accuracy significantly elevate a user’s perception of overall device capability. However, a critical structural disconnect emerged: Training (*β* = −0.024, *t*-value = 0.377, rejecting H5b), Health Information Accuracy (β = 0.015, *t*-value = 0.296, rejecting H6b), and device Functionality (β = −0.089, *t*-value = 1.130, rejecting H7a) all exhibited completely non-significant direct effects on ultimate User Acceptance (UA).

This uniform lack of direct significance is explained by re-conceptualizing technical parameters through Herzberg’s Motivation-Hygiene Theory. In chronic care environments, data accuracy, technical training, and system functionality do not operate as active, variance-driving motivators. A chronic patient does not actively choose to adopt a life-sustaining IoMT device because it is functional or accurate; rather, they treat absolute technical precision as a passive, non-negotiable hygiene factor. While its absence would trigger instantaneous rejection due to extreme physical hazard (e.g., a medical crisis triggered by a faulty sensor reading), its presence is fundamentally taken for granted as a baseline medical standard. Consequently, these elements fail to generate the statistical variance required to drive direct structural paths to behavioral intention.

#### 6.1.5 Awareness (AW)

Awareness (AW) of IoT devices was found to exert a highly significant positive impact on user acceptance (*β* = 0.162, *t*-value = 3.423, supporting H9). This finding strongly aligns with previous studies (52, 54), confirming that an individual’s explicit awareness of a device’s specific health-monitoring characteristics directly dictates their adoption tendency. When awareness increases, patients achieve a clearer comprehension of the device’s operational capabilities, which directly enhances its perceived clinical value. This demonstrates that transparency regarding how an IoMT tool actively captures and transmits biomedical telemetry empowers users, replacing technological intimidation with cognitive certainty.

#### 6.1.6 Risk (R)

The strong negative effect of Perceived Risk (R) on user acceptance was empirically validated (β = −0.170, *t*-value = 2.659, supporting H10). This finding supports a vast body of literature identifying risk as a primary barrier to technology adoption by Alraja and Liu et al. (42, 54), while contradicting outliers that reported small or non-significant effects (53, 85) This negative path highlights that when chronic patients experience ambiguity or perceive potential vulnerabilities—particularly regarding physical safety hazards like unauthorized device tampering or telemetry corruption—they instantly become resistant to adopting the technology. This underlines the fact that while data privacy can be psychologically bypassed by utility, explicit security risks that threaten physical well-being remain a powerful barrier to adoption.

#### 6.1.7 Innovativeness (IN)

This study confirmed a significant positive relationship between Personal Innovativeness (IN) and User Acceptance (UA) (*β* = 0.164, *t*-value = 3.813, supporting H11). This finding reinforces established research indicating that highly innovative individuals are naturally more receptive to embracing cutting-edge healthcare technologies (52, 58). In the IoMT context, naturally curious and innovative patients actively seek out advanced tracking features and efficiency, viewing emerging technologies as empowering tools for independence rather than complex operational burdens. This early-adopter sub-demographic provides a vital social foundation for the initial rollout of novel health solutions.

#### 6.1.8 User acceptance (*UA*)

In conclusion, this study explored the factors influencing the acceptance of IoT devices by patients with chronic diseases. Initially, the analysis posited that 11 factors, articulated through 19 hypotheses the study’s findings revealed that of the 19 hypotheses, 14 received empirical support, Interestingly, of the 11 factors identified, only six, namely, awareness (*AW*), innovativeness (*IN*), perceived severity (*PS*), perceived vulnerability (*PV*), risk (*R*), and regular compliance (*RC*), were found to significantly impact IoT acceptance.

### 6.2 Comparison of ANN results and SEM results

This study utilized an advanced two-stage hybrid PLS-SEM analysis to capture both linear and non-linear behavioral interactions. As illustrated in Table 10, the sensitivity analysis generated a highly nuanced predictive hierarchy that differs from the linear PLS-SEM path rankings. Most notably, Regular compliance (RC) was the main and most robust predictor of user acceptance (UA) with a normalised importance of 100%, followed by risk (R) at 91.20% normalised importance, and subsequently by perceived severity (PS) at 79.80% normalised relevance. Awareness (AW) next had a normalized relevance of 75.20%, followed by perceived vulnerability (PV) at 71.70%. Finally, innovativeness (IN) had a normalised significance of 57.40%.

**Table 10:** Comparison of ANN results and SEM results.

Independent variables	SEM (path coefficient)	Ranked	ANN (normalized importance %)	Ranked
*IN*	0.164	4	57.40%	6
*AW*	0.162	5	75.20%	4
*PV*	0.161	6	71.70%	5
*RC*	0.206	2	100%	1
*PS*	0.229	1	79.80%	3
*R*	0.17	3	91.20%	2

This rank divergence highlights a critical methodological reality documented in advanced IS research (86, 87). While PLS-SEM is constrained by the assumption of linear, compensatory effects, neural networks automatically capture complex, non-compensatory, and non-linear mathematical patterns. Consequently, a predictor like Regulatory Compliance exhibits a strong linear effect, but its predictive power multiplies exponentially within the non-linear ANN because it interacts with and neutralizes other variables (such as Perceived Risk). This confirms that in the minds of chronic patients, institutional regulation is the foundational anchor that dictates the non-linear configuration of their final adoption decisions.

## 7 Contributions and implications

### 7.1 Theoretical contributions

From an academic perspective, this research provides several vital theoretical contributions to the fields of Information Systems, cybersecurity, and digital healthcare adoption. This study successfully bridges the parallel silos of Protection Motivation Theory (PMT) and Task-Technology Fit (TTF). It demonstrates that for vulnerable clinical populations, task-technology characteristics (accuracy and training) do not directly drive acceptance; instead, they function as background hygiene factors that shape the technical infrastructure, while threat and coping appraisals drive the final behavioral variance. By executing a hybrid PLS-SEM and ANN architecture, this study provides a methodological blueprint for capturing human behavioral intention. It proves that relying solely on linear structural modeling can underestimate the true predictive weight of institutional variables like regulatory compliance.

Unlike highly localized studies that focus exclusively on a single medical device or a solitary health condition (88). This model intentionally avoids software or hardware specificity. By evaluating general IoMT perceptions across a diverse spectrum of chronic conditions, these findings possess significantly higher generalizability, providing a robust baseline for future cyber-physical health research.

### 7.2 Implications for healthcare cybersecurity policy

The empirical dominance of Regulatory Compliance (100%ANN importance) and Perceived Risk (91.20%) yields critical, clear directives for national healthcare cybersecurity policymakers. Healthcare cybersecurity strategies must completely abandon the unrealistic expectation that individual patients should manage their own endpoint device security. Because chronic patients operate under an institutional trust umbrella and treat data security as an expected baseline default, the burden of security enforcement must be shifted entirely onto regulatory bodies.

National regulators—such as the Saudi National Cybersecurity Authority (NCA) and the Saudi Food and Drug Authority (SFDA)—must enforce strict, zero-trust hardware compliance certification standards before any IoMT device can legally enter the consumer medical market. Software architectures must mandate automated, background firmware cryptographic patching and end-to-end data encryption protocols that operate seamlessly without requiring any technical literacy or manual configuration by the patient.

### 7.3 Social and public health implications

From a broader social perspective, this study provides vital insights for reducing digital health disparities and designing effective public health interventions. Because our structural model proves that technical training (*β* = 0.024) has no direct impact on adoption variance, public health organizations should redirect funding away from basic technical literacy classes. Instead, resources should be funneled into targeted Awareness campaigns that demonstrate the direct link between continuous biometric telemetry tracking and long-term disease control, physical independence, and longevity. Our findings indicate that as patients become aware of secure, state-compliant IoMT devices, they actively disseminate this safety reassurance to their immediate social and familial circles. In highly collaborative cultural contexts like Saudi Arabia, creating transparent, visibly regulated device ecosystems allows family members to confidently assist patients in managing their health, establishing a secure, collective communal support network.

### 7.4 Research limitations and future work

Despite the study’s contributions to existing literature, it faces limitations requiring future attention. The empirical data was captured via a cross-sectional survey design, providing a static snapshot of patient perceptions. Because both the cybersecurity threat landscape and user trust profiles are highly dynamic, future research should implement longitudinal tracking designs to evaluate the long-term temporal stability of the Chronic Utility Paradox.

The sample was drawn entirely from chronic patients embedded within the highly centralized, state-subsidized healthcare infrastructure of Saudi Arabia. Consequently, the findings regarding the non-significance of individual privacy and trust are bound to this high-trust macro ecosystem and may not seamlessly generalize to decentralized, highly privatized, or multi-payer healthcare markets (e.g., the United States). Future studies should conduct cross-national comparative analyses to test these institutional boundaries.

This study aggregated data across various chronic conditions (e.g., diabetes, cardiovascular diseases) and diverse IoMT form factors (e.g., non-invasive wearable sensors, invasive implantable devices). Future research should employ multi-group structural analyses (PLS-MGA) to assess whether the acute physical safety hazards associated with life-sustaining, invasive hardware (such as pacemakers) trigger significantly different risk-appraisal paths compared to non-invasive tracking wearables.

## 8 Conclusion

This study aimed to determine the dual nature of cybersecurity as either a driver or a barrier in the widespread implementation of IoT devices in healthcare. It integrated PMT and the TTF model, identifying additional factors, 12 in total. The study sought to identify the factors influencing this specific demographic’s behavior toward IoT device usage. The study employed a hybrid methodology integrating SEM and ANN analyses to clarify how acceptance of IoT devices was fostered among patients, reflecting their reliance on such tools for convenience and independence. The study was conducted among patients with chronic diseases (*n* = 603) with the dataset collected using an online survey. By expanding traditional frameworks and utilizing advanced analytics, the study provided an extensive perspective on the combination of individual and technological factors in shaping behavior, emphasizing the transformative role of cybersecurity in redefining protocols within the IoT market. This is crucial for safeguarding the emerging demographic of patients poised to impact future healthcare markets through their dependence on connected health devices.

The study’s conclusion synthesizes its impact across theoretical, practical, and social domains, alongside an analysis of challenges and future recommendations. Ultimately, the research met all established objectives and provided comprehensive answers to the guiding questions.

## Data Availability

The original contributions presented in the study are included in the article/Supplementary material, further inquiries can be directed to the corresponding author.
