# Supplementary material for: Understanding the role of cybersecurity in the internet of things within the health sector: an empirical study
Source: Front Public Health. 2026 Jul 17;14:1851079. doi: 10.3389/fpubh.2026.1851079 (PMC13423918; doi:10.3389/fpubh.2026.1851079)
Supplement: Supplementary file 1 [file Supplementary_file_1.docx]

**APPENDIX**

###### **Table A1:**Measures and scales

| Construct | Item codes | Questions | Sources |
| --- | --- | --- | --- |
| Perceived severity | *PS1* | If I suffered through having little knowledge about self-care, it would be severe. | [89],[38] |
|  | *PS2* | If I suffered while monitoring my personal daily healthcare, it would be serious. |  |
|  | *PS3* | If I suffered from medical diseases, it would be significant. |  |
| Perceived vulnerability | *PV1* | IoT health devices could be vulnerable to security incidents. | [90] |
|  | *PV2* | IoT health devices could be susceptible to a security incident. |  |
|  | *PV3* | A security problem with my personal data could occur if I do not comply with all the security policies of IoT health devices. |  |
| Privacy | *PRV1* | I have concerns regarding how the data are stored in IoT health devices. | [45] |
|  | *PRV2* | I have concerns regarding who has access to the data in IoT health devices. |  |
|  | *PRV3* | I think I will be affected if the data are hacked from my IoT health devices. |  |
|  | *PRV4* | I think my medical record can be held against me in cases of data leakage from IoT health devices. |  |
| Regular compliance | *RC1* | I believe that the law governs and interprets the practices of how IoT health device providers collect, use, and protect my private personal data. | [43],[91],[92] |
|  | *RC2* | Our hospital has a clear objective, strategy, and plan for adopting IoT health device systems. |  |
|  | *RC3* | Our hospital has a specific time frame for IoT health systems’ adoption. |  |
|  | *RC4* | I think government regulations encourage the adoption of new information technology (e.g., IoT for quality patient care). |  |
|  | *RC5* | I think the government can support safeguarding security and privacy concerns while using IoT health devices. |  |
| Training | *TRA1* | I have regular training in IoT health devices or have an associated qualification. | [47][46] |
|  | *TRA2* | I have learnt about IoT health devices in a formal hospital training course. |  |
|  | *TRA3* | If people are provided with proper training, then I think they would use IoT health devices |  |
| Health information accuracy | *HIA1* | The health information provided by the IoT health device is accurate. | [58] |
|  | *HIA2* | The health information provided by the IoT health device is trustworthy. |  |
| Functionality | *FN1* | In helping to monitor my health, the functions of the IoT health devices are enough. | [82] |
|  | *FN2* | In helping to complete my tasks, the functions of the IoT health devices are appropriate. |  |
|  | *FN3* | In general, the functions of the IoT health devices fully meet my needs, whether I am, or am not, in an emergency. |  |
| Trust | *T1* | I would trust the IoT health devices. | [93] |
|  | *T2* | I think the IoT health devices would be reliable. |  |
|  | *T3* | I believe that the IoT health devices would be employed in my best interests. |  |
|  | *T4* | I feel fine using the IoT health devices as they are generally reliable and accurate. |  |
| Awareness | *AW1* | I have a sufficient understanding of the threats that IoT health devices currently pose to my health. | [94] |
|  | *AW2* | I follow news and developments about security related to IoT health devices |  |
|  | *AW3* | I have a good understanding of IoT health devices. |  |
| Risk | *R1* | IoT health devices can have serious physical and psychological effects on individuals. | [95] |
|  | *R2* | IoT health devices can cause serious socio-economic losses. |  |
|  | *R3* | IoT health devices can take a heavy financial toll. |  |
|  | *R4* | IoT health devices can threaten my health and life. |  |
|  | *R5* | IoT health devices can affect my daily life. |  |
| Innovativeness | *IN1* | I am among the first to try out IoT health devices. | [96] |
|  | *IN2* | When I hear about a new IoT health device, I look for ways to adopt it. |  |
|  | *IN3* | I like to experiment with new IoT health devices. |  |
|  | *IN4* | I am a person who searches for novel approaches to help maintain my health. |  |
| User acceptance (behavioral intention) | *UA1* | I intend to use a healthcare IoT device in the future. | [97][38] |
|  | *UA2* | I intend to use a healthcare IoT device at every opportunity in the future. |  |
|  | *UA3* | I plan to increase my use of healthcare IoT devices in the future. |  |

######

###### **Table A2** : Cross-loadings

|  | *AW* | *FN* | *HIA* | *IN* | *PRV* | *PS* | *PV* | *R* | *RC* | *T* | *TRA* | *UA* |
| --- | --- | --- | --- | --- | --- | --- | --- | --- | --- | --- | --- | --- |
| *AW1* | 0.807 | 0.224 | 0.312 | 0.361 | 0.150 | 0.256 | 0.313 | 0.249 | 0.314 | 0.252 | 0.228 | 0.369 |
| *AW2* | 0.867 | 0.228 | 0.235 | 0.281 | 0.201 | 0.272 | 0.223 | 0.296 | 0.365 | 0.201 | 0.221 | 0.405 |
| *AW3* | 0.898 | 0.269 | 0.285 | 0.430 | 0.233 | 0.344 | 0.330 | 0.416 | 0.353 | 0.312 | 0.304 | 0.520 |
| *FN1* | 0.270 | 0.928 | 0.482 | 0.213 | 0.630 | 0.269 | 0.240 | 0.516 | 0.314 | 0.681 | 0.551 | 0.286 |
| *FN2* | 0.237 | 0.926 | 0.520 | 0.217 | 0.627 | 0.236 | 0.207 | 0.476 | 0.303 | 0.678 | 0.581 | 0.271 |
| *FN3* | 0.275 | 0.911 | 0.458 | 0.171 | 0.645 | 0.232 | 0.191 | 0.501 | 0.259 | 0.669 | 0.552 | 0.282 |
| *HIA1* | 0.333 | 0.496 | 0.949 | 0.272 | 0.382 | 0.270 | 0.265 | 0.275 | 0.324 | 0.424 | 0.630 | 0.310 |
| *HIA2* | 0.276 | 0.507 | 0.948 | 0.236 | 0.315 | 0.254 | 0.255 | 0.247 | 0.329 | 0.407 | 0.592 | 0.280 |
| *IN1* | 0.363 | 0.154 | 0.253 | 0.814 | 0.105 | 0.232 | 0.208 | 0.260 | 0.151 | 0.169 | 0.191 | 0.357 |
| *IN2* | 0.333 | 0.174 | 0.242 | 0.826 | 0.165 | 0.208 | 0.136 | 0.327 | 0.212 | 0.202 | 0.191 | 0.375 |
| *IN3* | 0.354 | 0.211 | 0.177 | 0.850 | 0.139 | 0.312 | 0.307 | 0.349 | 0.220 | 0.266 | 0.241 | 0.400 |
| *PRV1* | 0.238 | 0.588 | 0.288 | 0.148 | 0.878 | 0.235 | 0.177 | 0.554 | 0.242 | 0.623 | 0.391 | 0.319 |
| *PRV2* | 0.177 | 0.633 | 0.306 | 0.164 | 0.921 | 0.268 | 0.197 | 0.560 | 0.231 | 0.570 | 0.440 | 0.283 |
| *PRV3* | 0.208 | 0.635 | 0.394 | 0.134 | 0.899 | 0.278 | 0.293 | 0.568 | 0.293 | 0.617 | 0.412 | 0.340 |
| *PS1* | 0.271 | 0.219 | 0.247 | 0.235 | 0.244 | 0.872 | 0.333 | 0.321 | 0.337 | 0.271 | 0.277 | 0.474 |
| *PS2* | 0.348 | 0.256 | 0.248 | 0.282 | 0.274 | 0.874 | 0.338 | 0.329 | 0.340 | 0.305 | 0.334 | 0.497 |
| *PS3* | 0.249 | 0.199 | 0.203 | 0.257 | 0.216 | 0.798 | 0.258 | 0.263 | 0.289 | 0.251 | 0.238 | 0.389 |
| *PV1* | 0.324 | 0.168 | 0.215 | 0.237 | 0.180 | 0.310 | 0.901 | 0.340 | 0.405 | 0.263 | 0.290 | 0.456 |
| *PV2* | 0.280 | 0.238 | 0.269 | 0.256 | 0.251 | 0.323 | 0.923 | 0.339 | 0.410 | 0.326 | 0.318 | 0.456 |
| *PV3* | 0.324 | 0.226 | 0.268 | 0.230 | 0.249 | 0.375 | 0.918 | 0.368 | 0.397 | 0.321 | 0.334 | 0.470 |
| *R1* | 0.302 | 0.471 | 0.224 | 0.356 | 0.527 | 0.295 | 0.308 | 0.850 | 0.247 | 0.528 | 0.325 | 0.465 |
| *R2* | 0.346 | 0.482 | 0.290 | 0.330 | 0.539 | 0.315 | 0.337 | 0.852 | 0.298 | 0.532 | 0.358 | 0.456 |
| *R3* | 0.299 | 0.371 | 0.180 | 0.274 | 0.483 | 0.302 | 0.320 | 0.826 | 0.346 | 0.494 | 0.313 | 0.406 |
| *R4* | 0.330 | 0.471 | 0.217 | 0.294 | 0.527 | 0.291 | 0.308 | 0.803 | 0.307 | 0.543 | 0.319 | 0.382 |
| *RC2* | 0.377 | 0.298 | 0.292 | 0.214 | 0.275 | 0.332 | 0.387 | 0.332 | 0.891 | 0.295 | 0.359 | 0.474 |
| *RC3* | 0.294 | 0.265 | 0.291 | 0.175 | 0.177 | 0.316 | 0.385 | 0.287 | 0.870 | 0.255 | 0.308 | 0.436 |
| *RC4* | 0.373 | 0.264 | 0.317 | 0.225 | 0.287 | 0.347 | 0.383 | 0.315 | 0.854 | 0.267 | 0.320 | 0.471 |
| *T1* | 0.271 | 0.647 | 0.386 | 0.248 | 0.600 | 0.260 | 0.283 | 0.561 | 0.267 | 0.868 | 0.456 | 0.350 |
| *T2* | 0.266 | 0.665 | 0.368 | 0.220 | 0.589 | 0.279 | 0.296 | 0.545 | 0.270 | 0.903 | 0.438 | 0.328 |
| *T3* | 0.281 | 0.677 | 0.419 | 0.238 | 0.627 | 0.345 | 0.301 | 0.600 | 0.316 | 0.898 | 0.472 | 0.371 |
| *T4* | 0.260 | 0.631 | 0.392 | 0.216 | 0.581 | 0.279 | 0.309 | 0.542 | 0.264 | 0.907 | 0.444 | 0.338 |
| *TRA1* | 0.273 | 0.573 | 0.569 | 0.223 | 0.448 | 0.309 | 0.342 | 0.404 | 0.360 | 0.498 | 0.916 | 0.307 |
| *TRA2* | 0.246 | 0.548 | 0.580 | 0.253 | 0.413 | 0.295 | 0.332 | 0.362 | 0.346 | 0.464 | 0.917 | 0.328 |
| *TRA3* | 0.295 | 0.545 | 0.615 | 0.210 | 0.398 | 0.316 | 0.265 | 0.313 | 0.328 | 0.423 | 0.902 | 0.303 |
| *UA1* | 0.472 | 0.250 | 0.318 | 0.401 | 0.314 | 0.462 | 0.445 | 0.415 | 0.469 | 0.315 | 0.332 | 0.862 |
| *UA2* | 0.391 | 0.306 | 0.250 | 0.347 | 0.317 | 0.414 | 0.462 | 0.474 | 0.458 | 0.379 | 0.248 | 0.861 |
| *UA3* | 0.473 | 0.241 | 0.247 | 0.440 | 0.286 | 0.528 | 0.415 | 0.458 | 0.459 | 0.326 | 0.315 | 0.896 |

Reference

89. Sun Y, Wang N, Guo X, Peng Z. Understanding the acceptance of mobile health services: a comparison and integration of alternative models. *J Electron Commer Res*. (2013) 14:183.

90. Al-Sharafi MA, Al-Qaysi N, Iahad NA, Al-Emran M. Evaluating the sustainable use of mobile payment contactless technologies within and beyond the COVID-19 pandemic using a hybrid SEM-ANN approach. *Int J Bank Mark*. (2022) 40:1071–95. doi: 10.1108/IJBM-07-2021-0291

91. Guerra K, Koh C, Prybutok V, Johnson V. WIoT adoption among young adults in healthcare crises. *J Comput Inf Syst*. (2023) 63:1316–31. doi: 10.1080/08874417.2022.2150911

92. Abuabid A. A. “Preliminary model on factors influencing the adoption of medical internet of things (MIoT) Systems in Small and Medium-sized Hospitals in Saudi Arabia,” *Proceedings - 2024 3rd International Conference on Computational Modelling, Simulation and Optimization, ICCMSO 2024 Institute of Electrical and Electronics Engineers Inc*. (2024). 127–133.

93. Skalkos A, Stylios I, Karyda M, Kokolakis S. Users’ privacy attitudes towards the use of behavioral biometrics continuous authentication (BBCA) technologies: a protection motivation theory approach. *J Cybersecurity Privacy*. (2021) 1:743–66. doi: 10.3390/jcp1040036

94. Garcia-Perez A, Cegarra-Navarro JG, Sallos MP, Martinez-Caro E, Chinnaswamy A. Resilience in healthcare systems: cyber security and digital transformation. *Technovation*. (2023) 121:102583. doi: 10.1016/j.technovation.2022.102583

95. Yang C, Li J, Feng X, Su S, Zeng Q. Family protection motivation and economic vulnerability: a network analysis of public influenza risk perception, education and mitigation strategies in China. *Front Public Health*. (2025) 13:1633541. doi: 10.3389/fpubh.2025.1633541

96. Clegg C, Unsworth K, Epitropaki O, Parker G. Implicating trust in the innovation process. *J Occup Organ Psychol*. (2002) 75:409–22. doi: 10.1348/096317902321119574

97. Wixom BH, Todd PA. A theoretical integration of user satisfaction and technology acceptance. *Research*. (2005) 16:85–102. doi: 10.1287/isre.l050.0042
